# Acute Pancreatitis Accelerates Initiation and Progression to Pancreatic Cancer in Mice Expressing Oncogenic Kras in the Nestin Cell Lineage

**DOI:** 10.1371/journal.pone.0027725

**Published:** 2011-11-28

**Authors:** Catherine Carrière, Alison L. Young, Jason R. Gunn, Daniel S. Longnecker, Murray Korc

**Affiliations:** 1 Department of Medicine, Dartmouth Medical School, Hanover, New Hampshire, United States of America; 2 Department of Pathology, Dartmouth Medical School, Hanover, New Hampshire, United States of America; 3 Department of Pharmacology and Toxicology, Dartmouth Medical School, Hanover, New Hampshire, United States of America; 4 Norris Cotton Cancer Center, Dartmouth-Hitchcock Medical Center, Lebanon, New Hampshire, United States of America; Penn State Hershey Cancer Institute, United States of America

## Abstract

Targeting of oncogenic Kras to the pancreatic Nestin-expressing embryonic progenitor cells and subsequently to the adult acinar compartment and Nestin-expressing cells is sufficient for the development of low grade pancreatic intraepithelial neoplasia (PanIN) between 2 and 4 months. The mice die around 6 month-old of unrelated causes, and it is therefore not possible to assess whether the lesions will progress to carcinoma. We now report that two brief episodes of caerulein-induced acute pancreatitis in 2 month-old mice causes rapid PanIN progression and pancreatic ductal adenocarcinoma (PDAC) development by 4 months of age. These events occur with similar frequency as observed in animals where the oncogene is targeted during embryogenesis to all pancreatic cell types. Thus, these data show that oncogenic Kras-driven PanIN originating in a non-ductal compartment can rapidly progress to PDAC when subjected to a brief inflammatory insult.

## Introduction

Pancreatic ductal adenocarcinoma (PDAC) accounts for over 95% of all exocrine pancreatic malignancies and is the fourth leading cause of cancer-related death in the United States, with a median survival of 6 months [1]. In the last 30 years, patient survival rates have not improved substantially, and PDAC remains intractable in its late stages. Thus, there is an urgent need to develop new approaches for early detection of PDAC as well as new therapies targeting early cancer stages. Mouse models of PDAC that recapitulate human cancer have the potential to advance our understanding of the early events in cancer development.

**Figure 1:**
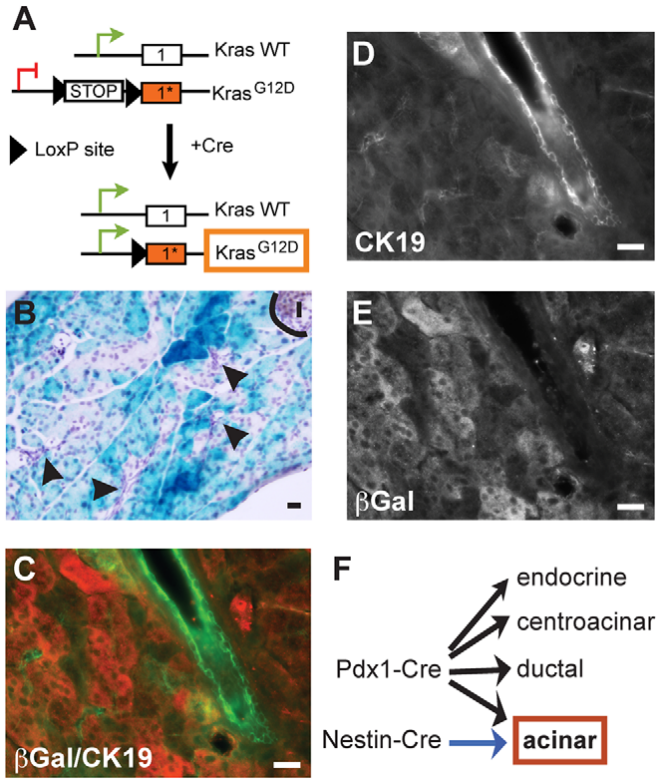
Targeting strategy with the Nestin-Cre transgene. (A) Schematic of the conditional Kras^G12D^ allele. In the absence of the Cre recombinase, only WT Kras is expressed; once Cre is expressed, the stop region is excised and Kras^G12D^ becomes expressed. (B–E) In Nestin-Cre/R26R mice, LacZ staining is observed only in acinar cells (B), and is absent in ductal cells (arrowheads) and islets (I). (C) Overlay of LacZ immunostaining (E) with CK19 (D) demonstrates that ductal cells are not targeted. (F) Pdx1-Cre leads to the expression of Kras^G12D^ in all pancreatic cell types while Nestin-Cre drives the expression of Kras^G12D^ only in acinar cells. Scale bar: 20 µm.

PDAC is now recognized to arise predominantly through progression of pancreatic intraepithelial neoplasia (PanIN), ranging from low- (PanIN-1A, -1B) to high-grades (PanIN-2, -3), PanIN-3 representing carcinoma *in situ* and the immediate precursor to ductal adenocarcinoma [2]. This histologic progression is correlated with the accumulation of genetic abnormalities of which mutations leading to the constitutive activation of KRAS are the earliest and most common (95%) [3]. The first relevant mouse models of PDAC were generated by targeting a conditionally mutated *Kras* allele (*Kras^G12D^*) to early pancreatic progenitors (using Pdx1 and Ptf1a promoters) and subsequently to all pancreatic cell types [4], [5]. These models recapitulated faithfully the full spectrum of human PanIN progression and pancreatic cancer development, demonstrating that oncogenic Kras expression is sufficient for PDAC initiation. To delineate the cells of origin of PDAC, several models were subsequently developed where mutated Kras was targeted to more restricted embryonic or adult cell populations. We used a Nestin-Cre driver to target Kras^G12D^ to acinar and Nestin-expressing cells (Nestin-Cre; LSL-Kras^G12D^ or N/K mice) (Fig. 1A), whereas others targeted oncogenic Kras to the acinar only or acinar/centroacinar cells with different elastase-Cre and mist1-Cre drivers [6], [7], [8], [9]. In all these models, PDAC initiation and progression could be observed, demonstrating that PDAC can have a non-ductal origin in mice. Although a recent study suggested that PDAC could initiate in the endocrine compartment, it has been shown that the promoter used in this study could also target a small fraction of acinar cells [10], [11]. In all the models where oncogenic Kras was activated during embryogenesis, low-grade PanINs developed, generally progressing over 8–12 months to high-grade lesions and PDAC. We were not able to observe progression to PDAC in N/K mice, as they generally die around age 6 months from of central nervous system complications.

Activation of Kras^G12D^ in the adult pancreas led to non-progressing low-grade lesion formation [7], [8], [12]. However, following induction of chronic pancreatitis, these animals developed high-grade lesions and progressed to PDAC [7]. These data are consistent with epidemiologic studies showing that patients suffering from chronic pancreatitis have a 16-fold increased risk of developing pancreatic cancer [13]. While brief episodes of acute pancreatitis (AP) lead to rapid PanIN progression and increased frequency of PDAC in Pdx1-Cre; LSL-Kras^G12D^ mice [14], [15], [16], it is unknown if it would also lead to PDAC in the Nestin-driven mouse model. We now show that N/K mice, when subjected to two episodes of caerulein-induced AP, rapidly develop high-grade PanINs that have the capacity to progress to PDAC. This study confirms that acute pancreatic injury has the potential to contribute to PDAC development in Kras-based mouse models and support the hypothesis that PDAC can arise in the Nestin cell lineage.

## Results

### Nestin progenitor cells do not give rise to ductal cells

We have previously shown that embryonic Nestin-expressing pancreatic progenitors give rise to acinar cells and not to endocrine or centroacinar cells, while a potential contribution to ductal cells was not completely excluded [6]. To resolve this issue, multiple lineage tracing experiments were done by breeding the mouse line that expresses the Cre recombinase under the control of Nestin regulatory elements [17] with the reporter mouse line R26R. This last line carries the β-Galactosidase (LacZ) gene under the control of the ubiquitous promoter R26 and a transcriptional stop region surrounded by LoxP sites (LoxP-Stop-LoxP or LSL) [18]. In the double transgenic mice, LacZ expression is activated following Cre-mediated recombination in embryonic Nestin-expressing cells (similar approach to the one described for Kras in Fig. 1A). Subsequently, by genetic transmission, all cells derived from the Nestin-expressing progenitors express LacZ. Sections across the pancreata of Nestin-Cre/R26R animals at 2 months of age were stained for LacZ activity. As observed before, the Nestin-Cre transgene allowed for the targeting of ∼70% of acinar cells (Fig. 1B). LacZ staining was observed only in acinar structures, never in the ductal, endocrine or centroacinar cells (Fig. 1B) and co-immunofluorescence with the ductal marker cytokeratin 19 (CK19) confirmed this observation (Fig. 1C–E). These results demonstrate that embryonic pancreatic Nestin-expressing progenitors do not give rise to ductal cells (Fig. 1F). Expression of LacZ was also rarely observed in pancreatic non-parenchymal cell types such as endothelial and mesenchymal cells (not shown).

### Acute pancreatitis dramatically accelerates lesion progression in N/K pancreata

**Figure 2:**
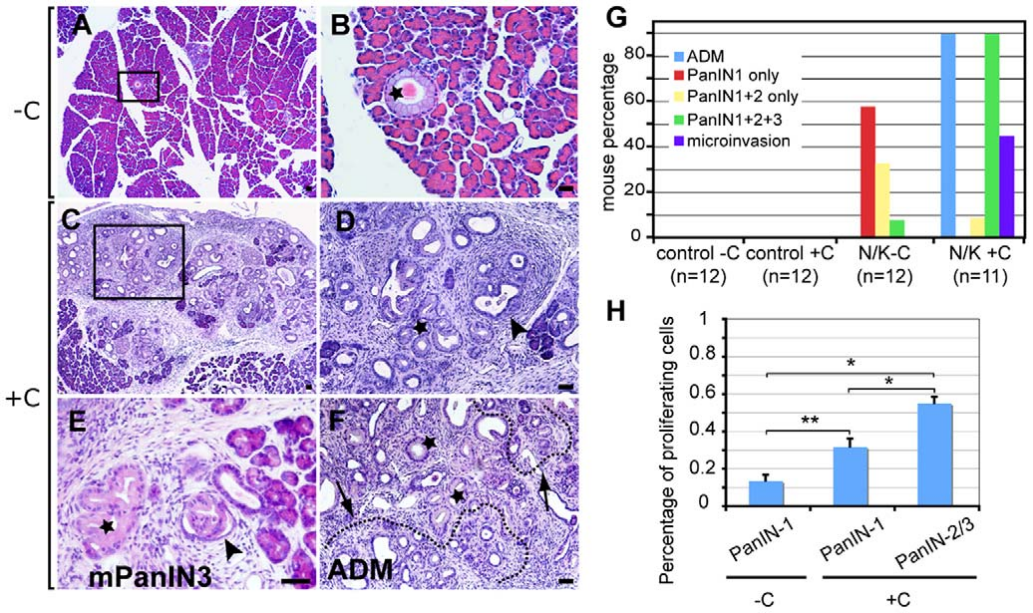
Caerulein treatment leads to major PanIN lesion progression 2 months following caerulein treatment. (A–F) H&E staining of 4 months old N/K pancreata: (A–B) untreated pancreas displaying rare low-grade lesions (*), (B) inset from (A) shows a single PanIN-1A lesion; (C–F) caerulein-treated pancreata show extensive dysplasia with a mix of low- (*) and high-grade (arrowhead) lesions (D, inset of C) and (E) as well as ADM foci (marked by dotted line)(F). (G) Morphometric analyses show dramatic increases in the number of animals displaying high grade PanIN lesions and areas of microinvasion in caerulein-treated pancreata. (H) Significant increases in proliferation are observed in caerulein treated pancreata (* P<0.05, ** P<0.01). Scale bar: 50 µm.

Between 2 to 6 months, N/K mice developed low grade PanINs in limited numbers (2–10 isolated lesions or small foci per section), as well as limited areas of acinar to ductal metaplasia (ADM) (Fig. 2A–B, G) similar to what is observed in Pdx1-Cre; LSL-Kras^G12D^ (P/K) mice [4], [14]. While with time P/K mice can develop PDAC but at very low frequency, N/K mutant mice rarely survived past 6 months due to severe neurological problems, preventing us from following a possible progression to pancreatic cancer.

We have previously shown that two episodes of acute pancreatitis (AP) induced by caerulein accelerated PanIN progression from low- to high-grade and increased PDAC frequency in the P/K mouse model [14]. To test the possibility that mutated Kras activation in the Nestin lineage could lead to PDAC, a cohort of 17 2-month-old N/K mice were subjected to caerulein treatment. Caerulein is a cholecystokinin (CCK) analog that binds to and activates the CCK receptor [19], [20]. While the drug dosage we used is low compared to the ones used by other groups [21], [22], extensive loss of acinar cells was observed in wild type mice at 24 and 48 hours following AP, concomitant with a dramatic inflammatory infiltration and interstitial expansion (Fig.S1) as described by others [21], [22], [23]. One week following AP, nearly complete regeneration of the pancreas was observed at the histological level, with rare residual inflammatory infiltrates while proliferation levels remained high as shown by Ki67 expression in acinar cells (Fig.S1A–D). These results are consistent with another published report [15]. To evaluate the possibility that following pancreatic injury, Nestin expression could be detected in different pancreatic cell types, short term lineage tracing experiments were done. LacZ staining in Nestin-Cre/R26R pancreata, 1 week post caerulein treatment, was observed only in acinar structures and no staining was detected in endocrine, centroacinar or ductal cells (not shown).

All 17 caerulein-treated N/K mice started getting sick when they were about 4 month-old, 2 months post-treatment, and had to be sacrificed. Six animals were lost without necropsy. By contrast, only 3 out of 9 untreated N/K mice died and none of the others were sacrificed due to sickness during this period. P/K mice when subjected to AP did not have shorter life expectancy than untreated mice at least over a period of 8 months (not shown). These observations suggested that the decrease in overall survival of N/K mice from 6 to 4 months was not directly related to pancreatic insufficiency. Four month-old N/K pancreata exhibited few PanIN-1A and -1B and rare ADM foci (less than 5% of the section) (Fig. 2A, B and G). Two months post caerulein-induced AP extensive pancreatic dysplasia was observed in N/K pancreata characterized by a mix of low- and high-grade PanIN and large ADM foci (Fig. 2C–F). The increase in lesion grade and the extensive ADM were documented by morphometric analyses that compared the presence and grade of lesions between sections from treated and untreated N/K mice. While most sections from untreated N/K pancreata exhibited low-grade lesions only and less than 5% of the section presented ADM, most caerulein-treated pancreata showed significant increases in PanIN-2 and -3 (P<0.0002) as well as extensive ADM covering 15 to 95% of the pancreas (Fig. 2G). These findings are similar to those we observed in a parallel cohort of P/K subjected at 2 month-old to caerulein-induced AP (Fig.S2); P/K mice were sacrificed 6 months post treatment (at age 8 months) which explains the higher rates of high-grade lesions and PDAC/microinvasion both in untreated and treated mice. As observed in N/K mice, all the caerulein-treated P/K mice show extensive ADM covering 25–95% of the sections (P<0.001) and dramatic progression to high-grade lesions and PDAC (Fig.S2). In contrast, in all control animals (wild type or carrying a single mutation, Nestin-Cre, Pdx1-Cre or LSL-Kras^G12D^ only) treated with caerulein, the pancreata appeared to be normal with occasionally residual traces of inflammation (not shown).

Low-grade lesions in N/K pancreata showed a proliferation index of 13%±3% similar to what is observed in P/K mice [4]. The high-grade lesions observed after caerulein treatment displayed as expected a high proliferation index (55%±3%), but interestingly low-grade lesions also showed a higher proliferation index of 31%±4% (Fig. 2H, S3A). Thus, the general increase in proliferation observed in the pancreas immediately following AP episodes [21] was maintained 2 months post injury but only in the neoplastic lesions in the presence of an activated Kras allele.

As observed in human PDAC samples, PanINs expressed high level of CK19, underscoring their epithelial nature, displayed mucus accumulation as shown by Muc5a expression (Fig.S3B–C), and were surrounded by collagen-rich stroma (Fig. S3F). The lesions showed a reactivation of the early pancreatic progenitor marker Pdx1 as well as the Notch signaling marker Hes1, both known to be reactivated in early stages of PDAC (Fig.S3D–E). ADM was demonstrated by the co-expression of acinar and ductal markers in the same cells (Fig.S3G–I) as observed by others [24]. These data confirm that caerulein-induced AP does not affect the nature of the lesions at the molecular level but accelerates their progression to high grade.

**Figure 3:**
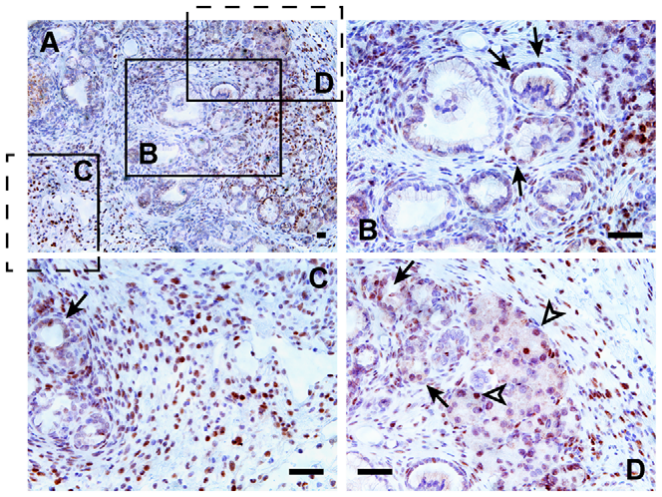
Stat3 activation is maintained 2 months post AP mainly in inflammatory cells and ADM area. (A–D) IHC for phospho-Stat3 in N/K pancreata 2 months post caerulein treatment. (A) Low magnification picture of a representative area. Phospho-Stat3 is rarely expressed in developing PanIN lesions but is observed in the cells that display a normal ductal morphology (B, arrows); (it is mainly expressed in inflammatory cells (C), in cells undergoing ADM (C, D, arrows) as well as in morphologically normal acinar cells (D, arrowheads). Scale bar: 20 µm.

It was shown recently that the inflammatory mediator Stat3 and the interleukin IL6 were essential contributors to PanIN progression and PDAC development at least in the early stages of the disease and were essential components for the acute response to caerulein-induced AP [16], [25]. Immunohistochemical analysis in N/K pancreata showed that phospho-Stat3 was still highly expressed 2 months post AP (Fig. 3) while IL6 was not detected (not shown). Phospho-Stat3 was shown previously to be highly activated in PanINs immediately following AP episodes [16], [25]. However, two months later phospho-Stat3 did not appear to be expressed in the lesions themselves but was abundantly detected in the inflammatory cells, in ADM foci and in groups of apparently normal acinar cells (Fig. 3A–D). Similarly, in untreated N/K mice, phospho-Stat3 was rarely detected and only in cells surrounding the PanINs (not shown).

### Oncogenic Kras activation in the Nestin cell lineage leads to PDAC development

**Figure 4:**
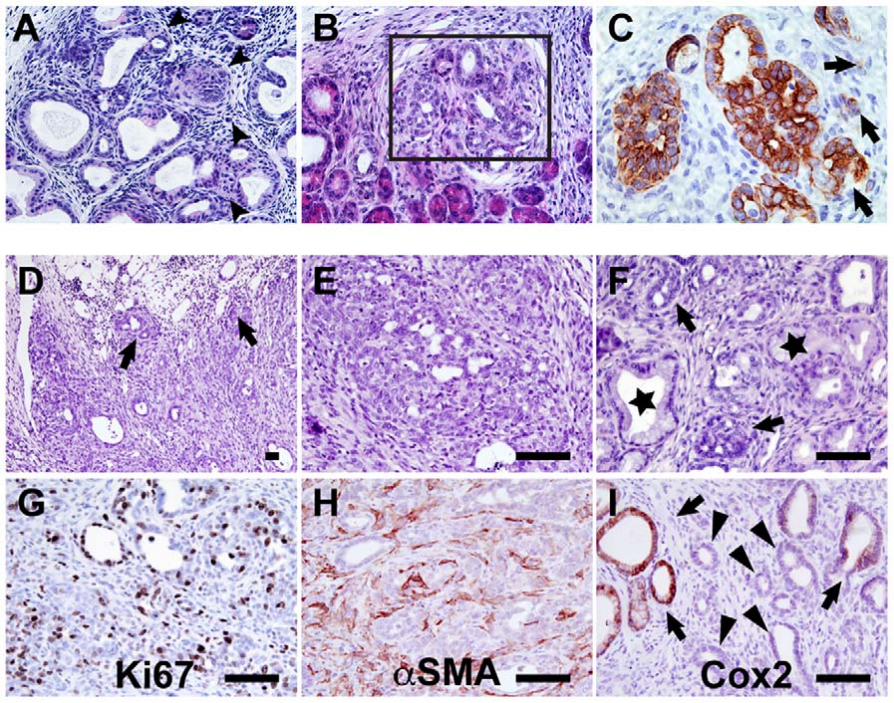
NK mice develop pancreatic ductal adenocarcinoma. High-grade lesions display microinvasion (A, arrowheads) and (B) as shown by CK19 expression (adjacent section) in cells in the adjacent stroma (C); (D) Low magnification shows the local invasion of fat and fibrous tissues (arrows). (E) Nuclei are enlarged, crowded and glandular morphology is maintained. (F) Low grade PanINs (stars) are observed in proximity with adenocarcinoma (arrows). (G) Adenocarcinoma cells are highly proliferative as shown by Ki67 expression. They are surrounded by reactive stroma characterized by SMA expression (H). Cox2 expression while high in the PanINs (arrows) is absent from the adenocarcinoma per se (arrowheads)(I). Scale bar: 20 µm.

In half the mice subjected to AP, areas of microinvasion can be observed in the pancreata as shown by the loss of the basement membrane in some PanIN-3 (Fig. 4A–B) and CK19 expression in cells within the adjacent stroma (Fig. 4C). From a small group of 4 N/K animals treated with caerulein at later stages (4 month-old), 3 survived until age 6 months and of these, 2 displayed tumors (diameter ∼5 mm) visible at the gross anatomical level. Only one tumor was available for histological analysis, displaying the morphological characteristic of PDAC, and revealing local invasion of the fat and fibrous tissues (Fig. 4D). Despite the fact that the nuclei were enlarged and crowded, a glandular morphology was maintained and low to high-grade PanINs were also observed (Fig. 4E–F). Large areas of the pancreas displayed extensive ADM (not shown). As observed in human PDAC, the tumors were highly proliferative as shown by Ki67 expression (Fig. 4G) and surrounded by conspicuously reactive stroma characterized by smooth muscle actin (SMA) expression (Fig. 4H). While we also observed high levels of Cox2 in PanINs, its expression was low in the cancer cells which is surprising as it is known to be upregulated in human PDAC (Fig. 4I).

### Nestin is expressed in human and mouse PanIN

**Figure 5:**
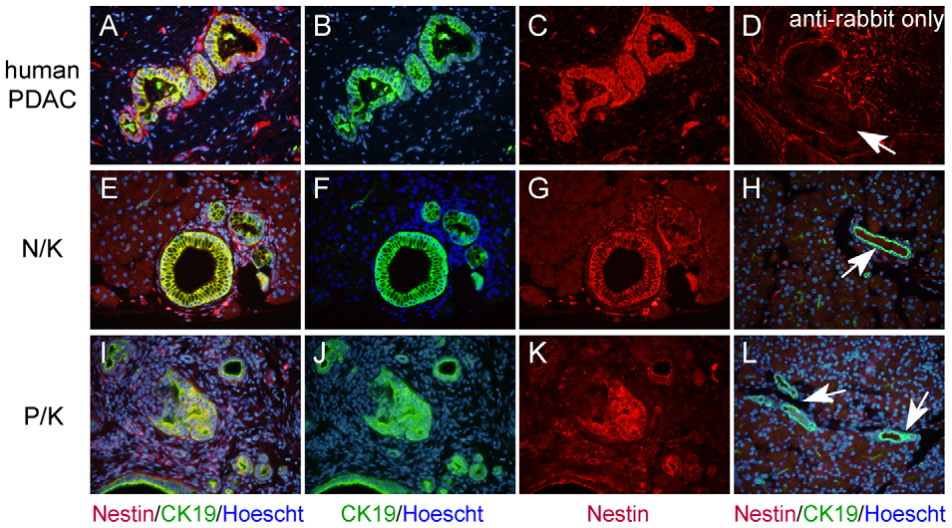
Nestin is expressed in human and mouse PanIN. (A–D) Human PanIN; mouse PanIN in NK (E–G) and PK (I–K). Overlays of CK19 (green), Nestin (red) and Hoescht (blue) show clear coexpression of Nestin with CK19 in the lesions (A, E, I). In contrast, in normal ducts, only CK19 is expressed (arrows)(H, L). (D) On human tissue, the secondary antibody alone generates high background in the stroma but no expression in PanIN (arrow). Scale bar: 20 µm.

Following AP, the pancreas undergoes intense proliferation and regeneration, and increases in Nestin expression have been observed by different groups [26], [27]. It is unclear if this increase occurs via an expansion of an already existing population of adult Nestin-expressing cells or is due to a transient expression of Nestin in dedifferentiating and proliferating acinar cells. We now show by immunohistochemistry that Nestin is consistently expressed in mouse PanIN from low to high-grade as well as in human PanINs and PDAC (Fig. 5).

## Discussion

We showed previously that N/K and the P/K mouse models develop low grade PanINs with similar timing and efficiency between 2 and 4 months of age [6]. These data suggested that PDAC initiation occurred mainly in the Nestin cell lineage, which consists in the pancreas of acinar cells and rare adult pancreatic Nestin-expressing cells. Still, the limited survival of the N/K mice past 6 months prevented us from pursuing the comparison to the PDAC stage. In the present study, we show that two close episodes of caerulein-induced AP in 2 month-old NK mice not only cause rapid PanIN progression from low- to high-grade but also with low frequency leads to PDAC. The progression efficiency appears similar again to the one observed in P/K mice following caerulein-induced AP [14]. These data demonstrate that N/K mice are a bona fide model of PDAC, and further support that the pancreatic Nestin lineage is the cell lineage that is mainly responsive to Kras mutation in the pancreas.

The targeting of oncogenic Kras to the acinar compartment, using different acinar-specific promoters also leads to the development in adult mouse of PanINs [8], [9] and infrequent invasive PDAC [7]. In N/K mice, Kras^G12D^ is activated in acinar cells as well as in adult Nestin-expressing cells. These later cells represent less than 0.1% of the total pancreas, appear integrated in the acinar structure but do not display differentiation markers (manuscript in preparation, CC and MK). At this stage, their function remains unknown. It is important to note that the Nestin regulatory elements used for these experiments target all Nestin-expressing cells in the animal [17]. Cre expression can be traced occasionally in endothelial and mesenchymal cells but there are no data in the literature suggesting that pancreatic cancer could initiate in non parenchymal cells. Thus taken together, these data provide strong evidence for a non-ductal cell origin of PDAC at least in the mouse.

Progression from PanIN to cancer is dramatically accelerated when the mice are subjected to environmental insults such as chronic pancreatitis [7], [10]. These data correlate with human epidemiological studies showing that chronic pancreatitis increases the risk of developing PDAC [13]. Our data and others show that caerulein-induced AP acts as cancer promoter in PDAC mouse models [14], [15], [16], suggesting that AP, when occurring in an individual whose pancreas harbors a Kras mutation, could potentially increase PDAC risk.

By which mechanisms does AP affect cancer progression? Recovery from AP in humans does not necessarily lead to irreversible damage and may be associated with pancreatic regeneration [28] as observed in the mouse. During the first 3 days following caerulein treatment, the mouse pancreas develop tubular structures in place of acini [23], and lineage tracing experiments using the elastase promoter suggest that part of these tubular structures are derived from acinar cells that appear to dedifferentiate, by going through a ductal-like metaplastic intermediate state before entering proliferation [23], [29]. New acinar cells originate then essentially from preexisting acinar cells [22], [23], [29] and have to undergo a secondary ductal to acinar metaplasia (DAM), an essential step for pancreas regeneration [15]. Interestingly, following AP, acinar cells that expressed oncogenic Kras died or underwent ADM. Among the acinar structures undergoing ADM, some appeared to give rise to PanIN, at least during the short period in which the mice were analyzed [15]. Pancreas regeneration also appeared to be stalled suggesting that Kras^G12D^-expressing cells that underwent ADM were not able to undergo the reverse process DAM to generate new acinar cells [15]. We observed extensive ADM/low-grade PanIN formation in both P/K and N/K pancreata in the 2–6 months following AP arguing again that large number of pancreatic cells respond to oncogene activation. Still, only few higher grade lesions can be detected. It suggests that while most acinar cells do respond to a certain extent to oncogenic Kras following caerulein-induced AP by undergoing ADM, they are not competent to progress to higher grade lesions and PDAC.

Nestin expression is upregulated in different models of pancreas regeneration and Nestin has been proposed as a transient marker of acinar dedifferentiation [26], [29]. It is also known as a stem cell marker during both embryogenesis and adulthood in different organs. One could then speculate that pancreatic adult Nestin-expressing cells are in fact adult acinar progenitors and that pancreas regeneration following AP occurs in part via the expansion of these progenitors. As such, adult Nestin-expressing cells would potentially be more competent for cancer initiation and progression following AP-induced proliferation, as suggested by the expression of Nestin in all grade lesions both in human and in mice. More experiments will be required to evaluate these possibilities.

Recent work showed that the inflammatory mediator Stat3 is activated at all stages of pancreatic cancer from low-grade PanIN to PDAC and is required for cancer progression [25], [30]. AP causes transient Stat3 activation in normal pancreas. In P/K mice, caerulein-induced AP leads to a major upregulation of phospho-Stat3 both in neoplastic lesions and the surrounding microenvironment in the 2–3 weeks following its induction. Indeed, phospho-Stat3 was shown to be directly responsible for the accelerated neoplastic progression in P/K mice following AP [16]. In our model two months post AP, phospho-Stat3 was only rarely detected in PanINs. In contrast, it was expressed mainly in the surrounding inflammatory cells, and in ADM foci as well as in small groups of histologically normal acinar cells. These observations suggest that Stat3 activation could be involved in metaplastic processes as the initiating steps in neoplastic transformation. The fact that extensive areas of ADM are still observed after 2 months also suggests that other events are required for the ADM to progress to PanIN. Increased levels of Kras^G12D^ activity could be an essential component for this progression [31], [32].

Recent studies showed that PDAC grows slowly over several decades and metastasize only in “late” stages [33], [34]. This suggests that early detection and prevention could be the most valuable approaches to confront this highly lethal cancer. Defining the cell types in which cancer can initiate as well as understanding the mechanisms by which environmental injuries can affect cancer progression are both essential for these approaches.

## Materials and Methods

All animal experiments were approved by the Institutional Animal Care and Use Committee at Dartmouth College, protocol number: 07-09-07.

### Mouse colony generation

The LSL-Kras^G12D^ (01XJ6—B6; 129-Kras2^tm4Tyj^) mice were generated by D.A. Tuveson and T. Jacks [35] and obtained from MMHCC, NCI. The Nestin-Cre mice (B6.Cg-Tg(Nes-cre)1^Kln/J^) generated by R. Klein [17] were purchased from the Jackson laboratory. Pdx1-Cre mice were a gift from G. Gu [35]. All genotyping were done by PCR following the conditions of the providers. Two episodes of acute pancreatitis were induced by a series of seven hourly intraperitoneal injections of caerulein given on 2 consecutive days, as described [14]. Caerulein (Sigma, St. Louis MO) was diluted in PBS and injected at a dose of 50 µg/kg of body weight. A second group of compound mutant and control animals received injections of PBS only. All animals were fasted for 12 hours before the experiment.

### Histology and immunohistochemistry

Mice were perfused with PBS then 10% formalin/PBS and the pancreata dissected. For βGalactosidase staining, tissues were transferred directly after perfusion into 30% sucrose at 4°C, and 24 hours later cryoembedded in OCT compound. For histology and most immunostaining, pancreata were fixed overnight and processed for paraffin embedding. Routine Hematoxylin and Eosin (H&E) staining was performed using standard procedures. For immunostaining, sections were deparaffinized, rehydrated and antigens were retrieved if required using a 2100-Retriever and antigen unmasking solution (Vector Laboratories). For cytokeratin 19 (CK19) immunostaining, Proteinase K antigen retrieval procedure was used. Immunostaining procedures were as described previously [5]. The antibodies and dilution used were: TromaIII (CK19 antibody developed by Rolf Kemler and obtained from Developmental Studies Hybridoma Bank, 1∶10), anti-βGalactosidase (rabbit, Cappel, 1∶200), anti-Muc5a (Novocastra, 1∶30), anti-Ki67 (mouse, Novocastra, 1∶200), anti-Hes1 (a gift from Dr. T. Sudo, Toray Inc., Kamakura, Japan; 1∶400), anti-Cox2 and anti-phospho-Stat3 (Tyr705) (both Cell Signaling 1∶50), anti-Pdx1 (Upstate1∶5000), anti-αSMA (Abcam, 1∶100), anti-Nestin (Chemicon, AB5922 for human, MAB353 for mouse, 1∶100), and anti-amylase (Calbiochem, 1∶500).

Pictures were captured with a QImaging EXI Blue camera and analyzed with ImagePro Plus 7.0. Fluorescence pictures were captured using the Black and White settings and artificially colored for analysis, except for Figure 5 in which pictures were taken using the color settings; H&E and immunochemistry pictures were captured using the Color settings. Photoshop was used to process the pictures.

## Supporting Information

Figure S1 **Caerulein treatment leads to acute pancreatitis.** H&E staining of pancreata untreated (A) and treated with caerulein (B, C). (B) 48 hours following caerulein treatment (48 h post C), the pancreas displays extensive ADM, exocrine atrophy and inflammatory infiltration. (C) At 1 week post C, the pancreas has regained its normal morphology but high levels of proliferation are still observed as shown by Ki67 expression (D).
(TIF)

Figure S2 **Morphometric analyses of P/K mice, 6 months post caerulein-induced AP.** Dramatic increases in the number of animals displaying extensive ADM, high grade lesions and PDAC/microinvasion areas are observed when compared with untreated animals.
(TIF)

Figure S3 **PanIN characterization in NK mice.** (A) Ki67 staining shows a clear increase in proliferation in low and high-grade lesions. High-grade PanINs express CK19 (B), Muc5a (C). Embryonic progenitors markers are reactivated (D, E). (F) Masson's trichrome blue staining shows the presence of collagen around PanIN lesions. (G) Extensive ADM is observed as shown by coexpression of CK19 (green, H) and amylase (red, I) in the same cells (white arrowheads). Scale bar: 20 µm.
(TIF)
