# Supplementary figures and images for: Acute Pancreatitis Accelerates Initiation and Progression to Pancreatic Cancer in Mice Expressing Oncogenic Kras in the Nestin Cell Lineage
Source: PLoS One. 2011 Nov 28;6(11):e27725. doi: 10.1371/journal.pone.0027725 (PMC3225359; doi:10.1371/journal.pone.0027725)

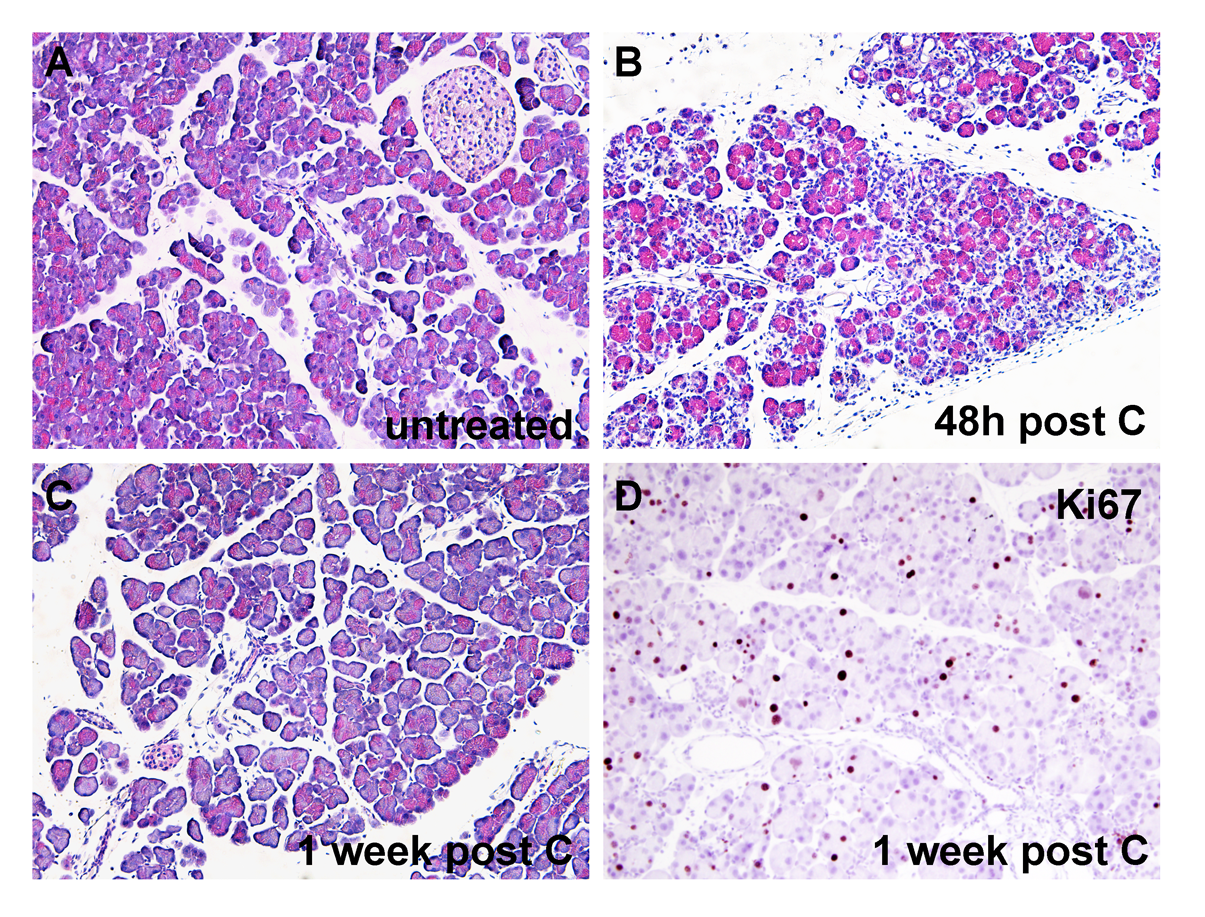

Supplement: Figure S1 — Caerulein treatment leads to acute pancreatitis. H&E staining of pancreata untreated (A) and treated with caerulein (B, C). (B) 48 hours following caerulein treatment (48 h post C), the pancreas displays extensive ADM, exocrine atrophy and inflammatory infiltration. (C) At 1 week post C, the pancreas has regained its normal morphology but high levels of proliferation are still observed as shown by Ki67 expression (D). (TIF) [file pone.0027725.s001.tif]

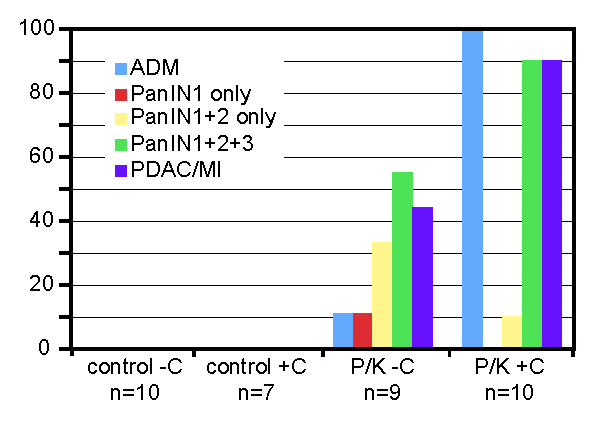

Supplement: Figure S2 — Morphometric analyses of P/K mice, 6 months post caerulein-induced AP. Dramatic increases in the number of animals displaying extensive ADM, high grade lesions and PDAC/microinvasion areas are observed when compared with untreated animals. (TIF) [file pone.0027725.s002.tif]

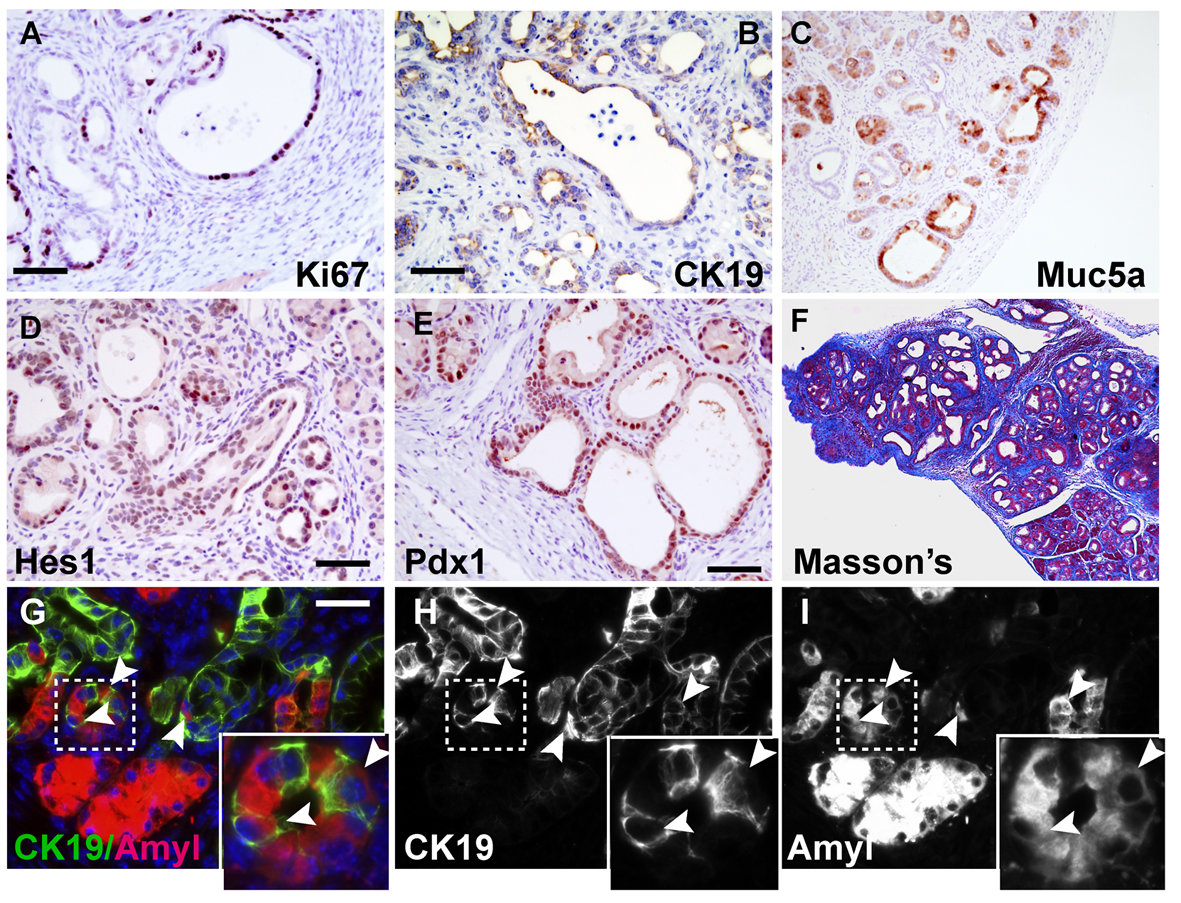

Supplement: Figure S3 — PanIN characterization in NK mice. (A) Ki67 staining shows a clear increase in proliferation in low and high-grade lesions. High-grade PanINs express CK19 (B), Muc5a (C). Embryonic progenitors markers are reactivated (D, E). (F) Masson's trichrome blue staining shows the presence of collagen around PanIN lesions. (G) Extensive ADM is observed as shown by coexpression of CK19 (green, H) and amylase (red, I) in the same cells (white arrowheads). Scale bar: 20 µm. (TIF) [file pone.0027725.s003.tif]
